# Random Bits Forest: a Strong Classifier/Regressor for Big Data

**DOI:** 10.1038/srep30086

**Published:** 2016-07-22

**Authors:** Yi Wang, Yi Li, Weilin Pu, Kathryn Wen, Yin Yao Shugart, Momiao Xiong, Li Jin

**Affiliations:** 1 Ministry of Education Key Laboratory of Contemporary Anthropology, Collaborative Innovation Center for Genetics and Development, School of Life Sciences, Fudan University, Shanghai 200433, China; 2 Unit on Statistical Genomics, Division of Intramural Division Programs, National Institute of Mental Health, National Institutes of Health, Bethesda, MD, USA; 3 Human Genetics Center, School of Public Health, University of Texas Houston Health Sciences Center, Houston, Texas, USA

## Abstract

Efficiency, memory consumption, and robustness are common problems with many popular methods for data analysis. As a solution, we present Random Bits Forest (RBF), a classification and regression algorithm that integrates neural networks (for depth), boosting (for width), and random forests (for prediction accuracy). Through a gradient boosting scheme, it first generates and selects ~10,000 small, 3-layer random neural networks. These networks are then fed into a modified random forest algorithm to obtain predictions. Testing with datasets from the UCI (University of California, Irvine) Machine Learning Repository shows that RBF outperforms other popular methods in both accuracy and robustness, especially with large datasets (N > 1000). The algorithm also performed highly in testing with an independent data set, a real psoriasis genome-wide association study (GWAS).

The most widely used methods for prediction include linear regressions, logistic regressions, *k*-Nearest Neighbors (*k*-NN)^1^, support vector machines (SVM)^2^, neural networks (NNs)^3^, extreme learning machines (ELM)^4^, deep learning (DL)^5^, random forests (RF)^6^,^7^, and generalized boosted models (GBM)^8^,^9^.

However, each method has its own drawbacks. For instance, linear regression and logistic regression handle linear and log-linear conditions, respectively, but may fail while dealing with nonlinear tasks. *k*-NNs are sensitive to the local structure of the data, with the best choice for *k* dependent on the properties of each datasets^10^. SVMs have uncalibrated class membership probabilities, large memory requirements (O(N^2^)), and difficult-to-interpret parameters^2^,^11^,^12^. NNs and DL are computationally expensive, with features learnt and tuned iteratively^13^,^14^. ELMs do not have sufficient features to handle complex works^15^. GBMs have high memory consumption and low evaluation speed^16^, as all base-learners must be evaluated in order to obtain predictions for the model. For RFs, decision trees are axis-parallel, which may lead to suboptimal trees; though oblique random forests provide one way to improve the performance of random forests^17^, ultimately they may fail on datasets with greater depth^18^.

We created Random Bits Forest (RBF), a classification and regression algorithm that integrates neural networks, boosting, and random forests. We compared the performance of RBF with that of seven other methods, using 28 datasets from the UCI (University of California, Irvine) Machine Learning Repository. We then tested RBF on real psoriasis genome-wide association study (GWAS) data.

## Methods

### Summary

**Figure 1:**
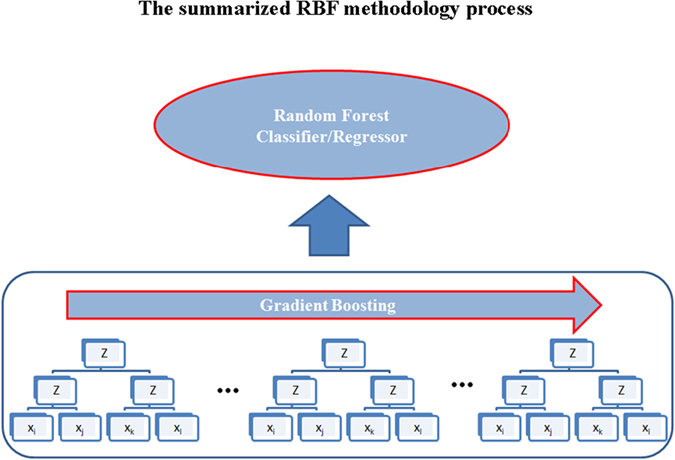
The summarized process. A 3-layer sparse neural network with random weights. ***Z*** represents threshold functions.

For clarity, features were standardized by subtracting the mean and dividing by standard deviation. The features were then transformed into random features/basis, by gradient boosting of the Random Bits base learner, a 3-layer sparse neural network with random weights, and fed to a random forest classifier/regressor to obtain predictions (Fig. 1).

### Random Bits

Our derived feature/basis/base learner is called Random Bits. It is a 3-layer sparse neural network with random weights. Two parameters were used to construct the neural network: *twist1* (the number of features connected to each hidden node) and *twist2* (the number of hidden nodes).

The features connected with hidden node are randomly assigned and interlayer weights are drawn from a standard normal distribution. The hidden nodes and the top node are the threshold units, with the threshold of each node determined by calculating the linear summation of its input for the *i*th sample z_i_ and choosing a random z_i_ among the sample as the threshold^15^.

### Boosting Random Bits

In order to generate many Random Bits, we used a gradient boosting scheme with the following pseudocode:

**For boost** = **1 to B**:

**For step** = **1 to S**:

     **1**: **residual = Y**; **MaxVar = 0**; **BestBit = NULL**;

     **2**: **For cand = 1 to C**:

          **1**: **Draw a random bit**, **RB**

          **2**: **Calculate the residual explained by RB**: **Var**

          **3**: **if** (**Var** > **MaxVar**) **{MaxVar = Var**; **BestBit = RB**;}

     **3**: **Set the random**_**bit**_**pool** [(**boost − 1**)** *** S + **step] = BestBit**

     **4**: **Mean[0] = E**(**residual|BestBit = 0**), **Mean[1] = E**(**residual|BestBit = 1**)

     **5**: **residual = residual − Mean[BestBit]**;

The algorithm launched *B* independent boosting chains, each with *S* steps. Each boosting chain undergoes the standard gradient boosting procedure, starting with a residual of *Y* and updating every step. In each step, *C* Random Bits features (*C* > 100) were generated, and the bit with the largest pseudo residual was chosen. The Random Bits from each independent boosting chain were collected to form a large (~10,000) feature pool. The Random Bits were stored in a compressed format requiring 1 bit per Random Bits per sample.

### Random Bits Forest

The produced Random Bits are eventually fed to Random Bits Forest. Random Bits Forest is a random forest classifier/regressor, but slightly modified for speed: each tree was grown with a bootstrapped sample and bootstrapped bits, the number of which can be tuned by users. The best bits among all the bootstrapped bits were chosen for each split. By making full use of the binary nature of Random Bits, through special coding and Streaming SIMD Extensions (SSE), acceleration was achieved, such that the modified random forest can afford ~10,000 binary features for large datasets (N = 500,000).

### Benchmarking

We benchmarked nine methods: linear regression (Linear), logistic regression (LR), *k*-Nearest Neighbors (kNN), neural networks (NN), support vector machines (SVM), extreme learning machines (ELM), random forests (RF), generalized boosted models (GBM), and Random Bits Forest (RBF). We used the RBF software available at http://sourceforge.net/projects/random-bits-forest/ and implemented the other eight methods using various R (v3.2.1) packages: stats, RWeka (v0.4-24), nnet (v7.3-8), kernlab (v0.9-19), randomForest (v4.6-10), elmNN (v1.0), and gbm (v2.1). We used ten-fold cross validation (accuracy, sensitivity, specificity and AUC) to evaluate each method’s performance. For methods sensitive to parameter selection, we manually tuned the parameters to obtain the best performance. As we chose the best handpicked parameters for each method respectively, the performance of each method based on the best parameters was comparable with each other. The results of tuning the parameters of sensitive methods on the real psoriasis genome-wide association study (GWAS) dataset were provided as Supplemental Materials 1. Benchmarking was performed on a desktop PC equipped with an AMD FX-8320 CPU and 32GB of memory. SVM, on some large-sample datasets, failed to complete benchmarking within reasonable time (1 week), so those results were left as blank.

### Benchmarked UCI Datasets Study

We benchmarked all datasets from the UCI Machine Learning Repository^19^ that fulfilled the following criteria including: (1) the dataset contains no missing values; (2) the dataset is in dense matrix form; (3) the dataset uses only binary classification; and (4) the dataset had clear instructions and specified the target variable.

We included 14 regression datasets (***3D Road Network***^20^, ***Bike Sharing***^21^***, Buzz in social media tomhardware***,***Buzz in social media twitter***,***Computer hardware***^22^, ***Concrete compressive strength***^23^,***Forest fire***^24^,***Housing***^25^,***Istanbul stock exchange***^26^,***Parkinsons telemonitoring***^27^,***Physicochemical properties of protein tertiary structure, Wine quality***^28^, ***Yacht hydrodynamics***^29^,***Year prediction MSD***)^30^ and 14 classification datasets (***Banknote authentication, Blood transfusion service center***^31^,***Breast cancer wisconsin diagnostic***^32^,***Climate model simulation crashes***^33^,***Connectionist bench***^34^,***EEG eye state, Fertility***^35^,***Habermans survival***^36^,***Hill valley with noise***^37^,***Indian liver patient***^38^,***Ionosphere***^39^,***MAGIC gamma telescope***^40^,***QSAR biodegradation***^41^,***Skin segmentation***)^42^.

### Applications on GWAS Dataset Study

We applied each method to a psoriasis genome-wide association (GWAS) genetic dataset^43^,^44^ to predict disease outcomes. We obtained the dataset, a part of the Collaborative Association Study of Psoriasis (CASP), from the Genetic Association Information Network (GAIN) database, a partnership of the Foundation for the National Institutes of Health. The data were available at http://dbgap.ncbi.nlm.nih.gov. through dbGaP accession number phs000019.v1.p1. All genotypes were filtered by checking for data quality^44^. We used 1590 subjects (915 cases, 675 controls) in the general research use (GRU) group and 1133 subjects (431 cases and 702 controls) in the autoimmune disease only (ADO) group. A dermatologist diagnosed all psoriasis cases. Each participant’s DNA was genotyped with the Perlegen 500K array. Both cases and controls agreed to sign the consent contract, and controls (≥18 years old) had no confounding factors relative to a known diagnosis of psoriasis.

**Figure 2:**
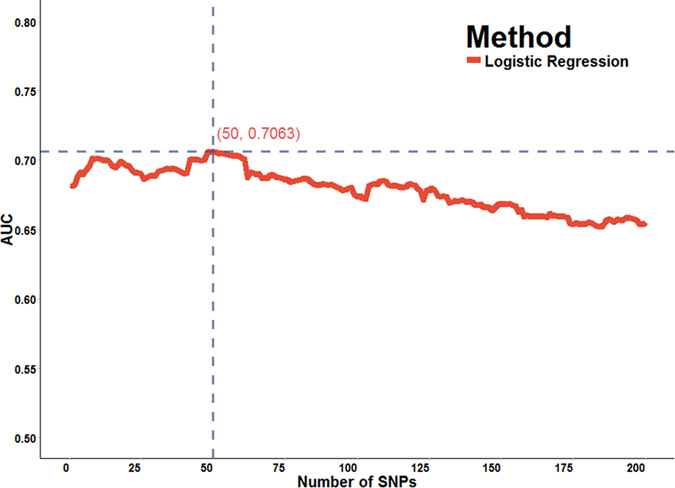
Maximum AUC of the independent ADO testing dataset with different numbers of markers.

We used both SNP ranking and multiple logistic regression methods, based upon allelic association p-values, for feature selection in training datasets and compared the different methods in both training and testing datasets. First, we trained the model based on the GRU dataset with different numbers of top associated SNPs, and then chose the robust and popular method (LR) to select the best number of SNPs as predictors based on the maximum AUC of the independent ADO (testing) dataset (Fig. 2 and Supplemental Materials 2). We then selected the best number (best number of SNPs = 50) of top associated SNPs as input variables and evaluated their performance in both the GRU (training) dataset and independent ADO (testing) dataset for each learning algorithm (except LR). To know more information of these selected 50 top associated SNPs, the Pearson’s R squared and Odds Ratio^45^ were also provided in Supplemental Materials 3.

**Figure 3:**
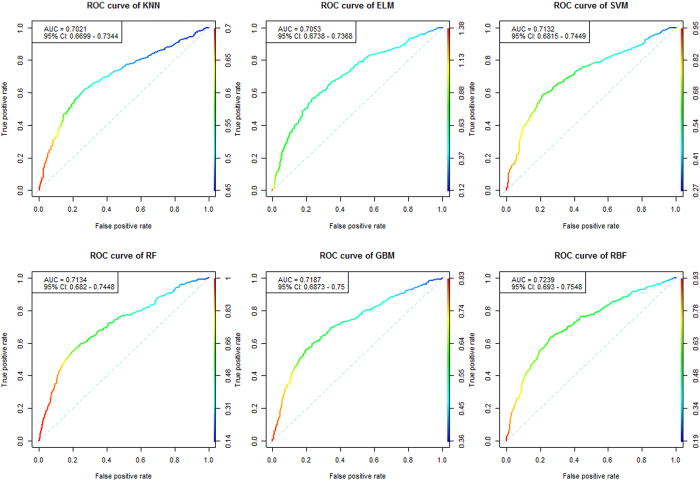
The ROC curve of six best benchmarked methods on the Psoriasis GWAS dataset of independent ADO group using selected best number of SNPs.

**Figure 4:**
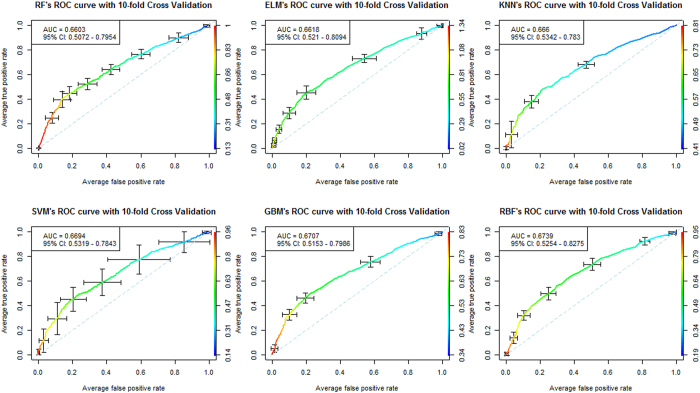
The average of ten-fold’s cross-validation ROC curve of six best benchmarked methods on the Psoriasis GWAS dataset of GRU group using selected best number of SNPs.

To evaluate a classification method’s performance on an imbalanced dataset, we used the area under the receiver operating characteristics (ROC) curve. The area under the curve (AUC) measures the global classification accuracy and is equal to the probability that a classifier will rank a randomly chosen positive instance higher than a randomly chosen negative instance^46^. We used the AUC as a measure of classifier performance for both GRU (training) and ADO (testing) datasets (Table 3, Figs 3 and 4). The 95% confidence interval (CI) of the AUC^47^, sensitivity, specificity and accuracy of all methods were also calculated by choosing the optimal threshold value.

## Results

### Results from UCI Datasets Study

Table 1 shows the regression root-mean-square error (RMSE) of all methods on 14 datasets. RBF was the top performing method in 13 and the second best performing method in 1. In the case (***Housing***) in which RBF was not the best method, the difference between RBF and the top performing method (RF) was within 2%. RF was the second best performing among the regression datasets. RBF’s performance exhibited the greatest improvement over that of the other methods with the ***3D Road Network*** dataset, a shallow task in which the methods predicted the altitude at specific points on a 3D map. However, RBF outperformed RF by allowing non-axis-parallel splitting.

Table 2 shows the classification error of each method among 14 datasets. RBF was the top performer in 8 datasets, the second best in 5, and the third best for 1. In the cases RBF was not the best method, the difference between RBF and the top performing method was within 2%. SVM was the second best method among classification datasets. RBF’s performance exhibited the greatest improvement over that of the other methods with the ***Hill valley with noise*** dataset, a deep task in which the methods classified the shape (“hill” or “valley”) of a time series with 100 time points. Although all other methods, except neural networks, failed to well perform this task, RBF and its 3-layer random neural network features worked well on this dataset.

Furthermore, we also observed that the datasets in which RBF performed best were all big datasets (N > 1000 with limited features, Table 1 and Table 2). This is due to the nature of trees, which inherently require larger samples than do regressions.

### Results from GWAS dataset study

Figure 2 and Supplemental Materials 2 shows that the ideal number of biomarkers for prediction of psoriasis was 50 in the efficient LR classifier. When the number of biomarkers was less than 20, the AUC of independent ADO (test) dataset was unstable in LR classifier. On the other hand, as the number of biomarkers approached 50, performance improved and stabilized: the best AUC for LR was 0.7063, respectively. Performance did not significantly improve as the number of biomarkers increased over 50.

As seen in Table 3, all benchmarked methods were used to construct effective diagnosis models for psoriasis prediction based on optimal number of SNP subsets. No significant unbalances were found in the training and testing datasets, suggesting the credibility and stability of the prediction models. The average of AUC of 10-fold cross-validation^48^ in the training dataset and AUC of the independent testing dataset were used to evaluate the performance of all methods. The AUC of each method ranged from 0.6192−0.6739 in the training dataset and from 0.6563−0.7239 in the testing dataset. We found that RBF, GBM, SVM and RF were the four top performing methods in both the training dataset and the testing dataset. RBF was the top performer in both the training dataset (AUC = 0.6739, 95% CI: [0.5254, 0.8275], sensitivity = 0.6317, specificity = 0.6490, accuracy = 0.6390) and the testing dataset (AUC = 0.7239, 95% CI: [0.6930, 0.7548], sensitivity = 0.6543, specificity = 0.7151, accuracy = 0.6920). The ROC curves for each method are also shown in Fig. 3 and Fig. 4 for performance comparison visualization.

Furthermore, RBF appeared to be robust in sensitivity and specificity in both the training and testing datasets. Although the sensitivity and specificity of RBF were not the best for all datasets, its AUC still was the top performer in both GRU (training) and ADO (testing) datasets. This characteristic of RBF is also applicable in the unbalanced dataset, whose prediction performance may be easily influenced by the disease population ratio. In Table 3, we see that although KNN has the second accuracy (accuracy = 0.6884) in the testing dataset, its AUC performance (AUC = 0.7021) is poor because it pays more attention to specificity (specificity = 0.7279) than sensitivity (sensitivity = 0.6241).

## Discussion

Random forests are among the top performing algorithms for machine learning, as they are accurate, fast, flexible, and mature. Random forest^6^ is a substantial modification of bagging which builds a large number of de-correlated trees and then averages the trees. The main idea of random forests is to improve the variance reduction of bagging by reducing the correlation between trees without increasing the variance heavily^49^. And the target is achieved in the tree-growing process by randomly selecting the input variables. Thus, Random Bits Forest mainly focuses on the automated feature engineering of random forests. We also obtain good results if we feed random bits to a regularized linear regression, though, in big data cases, no better than we get from random forests. And the statistical inference^50^ of random forests equally applies to RBF.

RBF outperforms the random forest algorithm by breaking its two limitations: the limitation to axis-parallel splitting that may lead to suboptimal trees^17^, and the decision tree depth of two that could fail on dataset with greater depth^18^. To overcome the first limitation, we used random projections. Because of pre-generation of many (~10,000) random projections, the tree is allowed to grow with more freedom. To overcome the second limitation, we improved naïve random projections with a 3-layer random neural network. We then defined a random neural network based on the original features and took its output as a derived feature/basis. Such additional depth may be crucial for specific datasets (UCI dataset: ***Hill valley with noise***, shown in Table 2).

Compared to oblique random forests, RBF generated non-axis parallel features before random forest while oblique random forests generates oblique splits within the tree-growing process. One crucial improvement to our random projections was to use 3-layer random neural networks as random projection/basis, giving the random forest more depth. Additional layers did not improve accuracy on the benchmarked datasets, potentially because 3-layer neural networks are already universal approximations.

In order to make full use of our ~10,000 bits budget, we need a feature selection procedure rather than naïve random projections. Feature selection was achieved by employing the gradient boosting framework. Instead of directly using the boosting predictions, we collected the boosted basis and fed them into the random forest. First, we found the random bit that best explained the residual and subtracted its effect from the residual to avoid highly correlated random bits. For the ***Hill valley with noise*** dataset, this method for feature selection reduced error from 11% to 2.5%, compared with naïve random projections.

In the boosting procedure, we used multiple independent boost chains, originally just for ease of parallel computing. However, multiple chains also reduced the local optimum problem and led to better prediction. For small datasets, 256 boost chains were used.

Large sample (N > 1000) are important for the success of RBF since trees are more flexible models than are linear models and as a result require a larger sample size. For smaller samples, regularization is useful, which was achieved by limiting the bootstrapped sample size. The consequence is that each tree was suboptimal and biased, but the trees are further decorrelated, thus reducing variance. Reducing feature bootstrap also helped to regularize the problem.

In summary, we firstly present Random Bits Forest (RBF), an original classification and regression algorithm that integrates the advantages of neural networks (for learning depth), boosting (for learning width), and random forests (for prediction accuracy). That is the reason why Random Bits Forest will perform better than other methods.

In conclusion, RBF is a novel robust method for machine learning, which is especially effective in datasets with large sample sizes (N > 1000). Our work indicates that RBF performs better if fed with extracted/selected features by using appropriate feature selection methods.

## Additional Information

**How to cite this article**: Wang, Y. *et al.* Random Bits Forest: a Strong Classifier/Regressor for Big Data. *Sci. Rep.* **6**, 30086; doi: 10.1038/srep30086 (2016).

## Supplementary Material

Supplemental Materials 1

Supplemental Materials 2

Supplemental Materials 3

## Figures and Tables

**Table 1:** Regression RMSE of all methods on 14 datasets.

Regression RMSE	Sample	Feature	Linear	KNN	NN	ELM	SVM	GBM	RF	RBF
***Computer hardware***	209	7	69.62	63.13	134.91	159.23	93.63	91.67	59.66	**58.39**
***Yacht hydrodynamics***	308	6	9.13	6.43	1.18	1.96	1.03	1.16	**1.00**	**1.00**
***Housing***	506	12	4.88	4.10	4.94	7.92	3.16	3.40	**3.07**	3.13
Forest fire*	517	13	1.50	**1.40**	2.10	**1.40**	1.50	**1.40**	1.41	**1.40**
***Istanbul stock exchange***	536	8	**0.01**	0.01	0.04	0.02	**0.01**	**0.01**	**0.01**	**0.01**
***Concrete compressive strength***	1030	9	10.53	8.28	6.36	13.18	5.25	4.72	4.53	**4.18**
***Parkinsons telemonitoring***	5875	19	9.74	6.10	6.69	10.35	6.02	2.10	1.65	**1.19**
***Wine quality***	6497	11	0.74	0.70	0.73	0.92	0.67	0.67	0.58	**0.57**
***Bike sharing***	17389	16	141.87	104.58	65.99	94.56	102.37	75.47	39.97	**38.26**
Buzz in social media tomhardware*	28179	97	1.45	0.76	0.37	1.58	1.49	**0.31**	**0.31**	**0.31**
***Physicochemical properties***	45730	9	5.19	3.79	6.12	6.12	4.16	5.05	3.45	**3.27**
***3D Road Network***	434874	2	18.37	6.44	15.55	16.95	12.53	14.82	3.86	**1.20**
***Year prediction MSD***	515345	90	9.55	9.22	10.93	11.47	—	9.63	9.24	**8.87**
Buzz in social media twitter*	583250	78	1.33	0.52	0.51	1.03	—	0.48	**0.47**	**0.47**

Bold: The bold means the first place result of all methods compared.

*The * means the dependent variable of the corresponding data was transformed by log function to be more asymptotically normal.

The best RBF’s RMSE was significantly less than the second best RF using Wilcoxon Matched-Pairs Signed-Ranks Test (*p-value* = 0.007185).

**Table 2:** Classification error of all methods on 14 datasets.

Classification error%	Sample	Feature	LR	KNN	NN	ELM	SVM	GBM	RF	RBF
***Fertility***	100	9	15.00	12.00	15.00	24.00	12.00	**12.00**	**12.00**	**12.00**
***Connectionist Bench***	208	60	26.00	13.02	21.67	14.43	**10.14**	12.52	12.52	12.02
***Habermans survival***	306	3	25.85	25.16	30.71	27.40	26.45	27.12	27.4	**25.12**
***Ionosphere***	351	34	10.26	10.25	11.98	10.28	5.13	6.26	6.55	**4.26**
***Climate Model Simulation Crashes***	540	18	**4.26**	7.04	5.56	5.93	4.44	5.74	6.48	4.81
***Breast Cancer Wisconsin Diagnostic***	569	30	5.09	2.81	8.45	8.80	**1.93**	3.33	2.98	2.28
***Indian Liver Patient***	579	10	27.83	27.82	30.21	28.34	28.51	27.47	**26.09**	26.42
***Blood Transfusion Service Center***	748	4	22.86	**19.65**	24.46	23.80	20.19	21.66	21.79	19.92
***QSAR biodegradation***	1055	41	13.37	13.75	14.98	22.38	12.14	12.89	12.42	**11.95**
***Hill valley with noise***	1212	100	42.00	45.71	5.28	23.42	34.73	43.89	40.50	**2.47**
***Banknote authentication***	1372	4	1.02	0.15	**0.00**	**0.00**	**0.00**	0.15	0.51	**0.00**
***EEG Eye State***	14980	14	35.75	15.37	31.57	42.34	19.52	8.46	5.96	**3.66**
***MAGIC Gamma Telescope***	19020	10	20.88	15.86	13.17	22.64	12.30	11.75	11.73	**10.36**

Bold: The bold means the first place result of all methods compared.

The best RBF’s error% was significantly less than the second best SVM using Wilcoxon Matched-Pairs Signed-Ranks Test (*p-value* = 0.04584).

**Table 3:** Psoriasis prediction performance with all methods based on best number of SNP subsets.

	Independent testing dataset (ADO dataset)					Training dataset (GRU dataset) with 10-fold cross validation*				
	Sensitivity	Specificity	Accuracy	AUC	95% CI of AUC	Sensitivity	Specificity	Accuracy	AUC	95% CI of AUC
NN	0.6404	0.5840	0.6055	0.6563	[0.6240, 0.6886]	0.5347	0.6657	0.5899	0.6192	[0.4388, 0.7893]
KNN	0.6241	0.7279	0.6884	0.7021	[0.6699, 0.7344]	0.6428	0.6553	0.6478	0.6660	[0.5342, 0.7830]
ELM	0.6589	0.6610	0.6602	0.7053	[0.6738, 0.7368]	0.6305	0.6403	0.6346	0.6618	[0.5210, 0.8094]
RF	0.6311	0.7051	0.6770	0.7134	[0.6820, 0.7448]	0.6036	0.6703	0.6314	0.6603	[0.5072, 0.7954]
SVM	0.6589	0.6952	0.6814	0.7132	[0.6815, 0.7449]	0.6569	0.6419	0.6503	0.6694	[0.5319, 0.7843]
GBM	0.6473	0.7080	0.6849	0.7187	[0.6873, 0.7500]	0.5890	0.7129	0.6415	0.6707	[0.5153, 0.7986]
RBF	0.6543	0.7151	0.6920	**0.7239**	[0.6930, 0.7548]	0.6317	0.6490	0.6390	**0.6739**	[0.5254, 0.8275]

Bold: The bold means the first place result of all methods compared. *AUC, sensitivity, specificity, and accuracy were its average value in 10-fold CV,

95% CI of AUC represents the range of the 95% CI of AUC in 10-fold CV.
